# Unveiling Neurovascular Orofacial Pain: An Underdiagnosed Form of Chronic Orofacial Pain

**DOI:** 10.3390/healthcare11121722

**Published:** 2023-06-12

**Authors:** Yaron Haviv

**Affiliations:** Faculty of Dental Medicine, Department of Oral Medicine, Sedation and Imaging, Hadassah Medical Center, Hebrew University of Jerusalem, Jerusalem 91120, Israel; yaronha@hadassah.org.il

Neurovascular orofacial pain (NVOP) is a relatively rare type of facial pain syndrome that shares similarities with migraine, orofacial migraine, and trigeminal autonomic cephalalgias (TACs). NVOP is characterized by pain in the orofacial region. Diagnosing NVOP can be particularly challenging. Recently, it was included as a recognized diagnosis in the latest classification of orofacial pain—ICOP [1]. 

In this editorial, we explore the symptoms, causes, diagnosis, and treatment of NVOP [2,3,4].

**Symptoms:**

NVOP typically presents as episodic, unilateral or bilateral pain or discomfort in the orofacial region. The pain is often described as a dull ache, pressure, or throbbing and may migrate between teeth. Additional symptoms can include sensitivity to light, sound, or odors; nausea; vomiting; visual disturbances; and numbness or tingling of the face, mouth, or tongue [5,6].

**Causes:**

The exact cause of NVOP is unknown, but it is thought to be related to neurovascular mechanisms that contribute to other types of migraines. These mechanisms involve changes in the blood vessels and nerves in the brain, resulting in inflammation, blood vessel dilation, and activation of pain pathways. NVOP can be triggered by various factors such as stress, hormonal changes, certain foods, weather or altitude changes, and physical exertion [5].

**Diagnosis:**

Diagnosing NVOP can be challenging due to its resemblance to other dental and facial pain disorders such as acute pulpitis, trigeminal neuralgia [7,8], or temporomandibular joint disorder [9,10]. A thorough medical history and physical examination are crucial for ruling out other potential causes of facial pain, such as dental problems or sinusitis. Imaging studies such as MRI or CT scans may be conducted to exclude other neurological conditions. The diagnosis of NVOP is typically based on characteristic symptoms and may require consultation with a headache specialist or neurologist [6].

**Treatment:**

The NVOP treatment approach usually involves a combination of lifestyle modifications, medications, and nonpharmacological therapies. Lifestyle modifications may include avoiding triggers, maintaining regular sleep patterns, and employing stress-reduction techniques such as meditation or yoga. Naturally, success depends on individuals’ expectations and coping strategies [11]. Medications commonly used to manage migraines can be effective for NVOP. For infrequent attacks (less than five per month), drugs that can be taken on an as-needed basis, such as triptans (migraine-specific medication) and NSAIDs such as ibuprofen, may be suitable. These medications must be taken at the onset of each attack for optimal pain relief. If NVOP becomes chronic, with more than four severe pain attacks per month, a preventive approach is recommended using medications such as beta blockers, antiepileptic drugs, antidepressants, or newer CGRP receptor antagonists specifically developed for migraine relief. Nonpharmacological therapies such as biofeedback, acupuncture, or physical therapy can also be beneficial in alleviating symptoms [12].

**In conclusion**, neurovascular orofacial pain or NVOP is a relatively rare type of facial pain syndrome that shares similarities with other facial pain disorders, including migraines. NVOP is characterized by episodic pain or discomfort in the orofacial region, which may migrate between the teeth. Treatment typically involves lifestyle modifications, medications, and nonpharmacological therapies. If you experience symptoms of NVOP, medical attention must be sought to exclude other potential causes and receive appropriate treatment.
